# P-2023. A Quality Improvement Initiative to Improve Sexual Orientation and Gender Identity (SOGI) Documentation in a Southern Academic HIV Clinic

**DOI:** 10.1093/ofid/ofaf695.2187

**Published:** 2026-01-11

**Authors:** Emily D Niehaus, Hayley Cunningham, Naseem Alavian, Lana Abusalem, Sarah Schmidt, Jaleesa Spears, Molly McDonough

**Affiliations:** Duke University School of Medicine, Durham, NC; Duke University School of Medicine, Durham, NC; 1Department of Medicine, Division of Infectious Diseases Duke University School of Medicine, Durham, North Carolina; Duke University School of Medicine, Durham, NC; Duke University Health System, Durham, North Carolina; Duke University Health System, Durham, North Carolina; Duke University School of Medicine, Durham, NC

## Abstract

**Background:**

Affirming patients' individual perspectives and life experiences is crucial for building trust and providing high-quality care. This is especially important for people with HIV infection (PWH) and sexual and gender minority (SGM) patients. Sexual orientation and gender identity (SOGI) data is underreported in structured electronic health record (EHR) fields. Our quality improvement project in the Duke Infectious Disease HIV Clinic aimed to improve SOGI documentation among new and established PWH in our clinic to 90% over 24 months.

**Figure 1.**
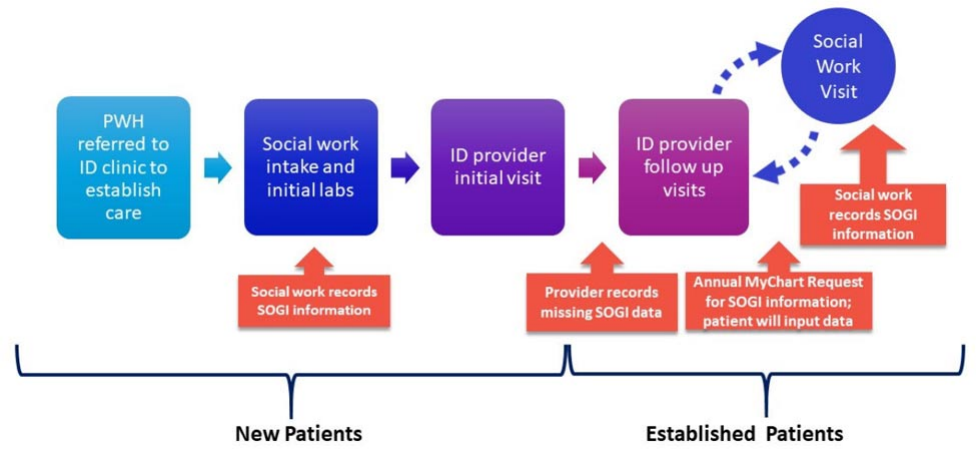
Process Map of Opportunities for SOGI documentation for New and Established Patients with HIV

**Figure 2.**
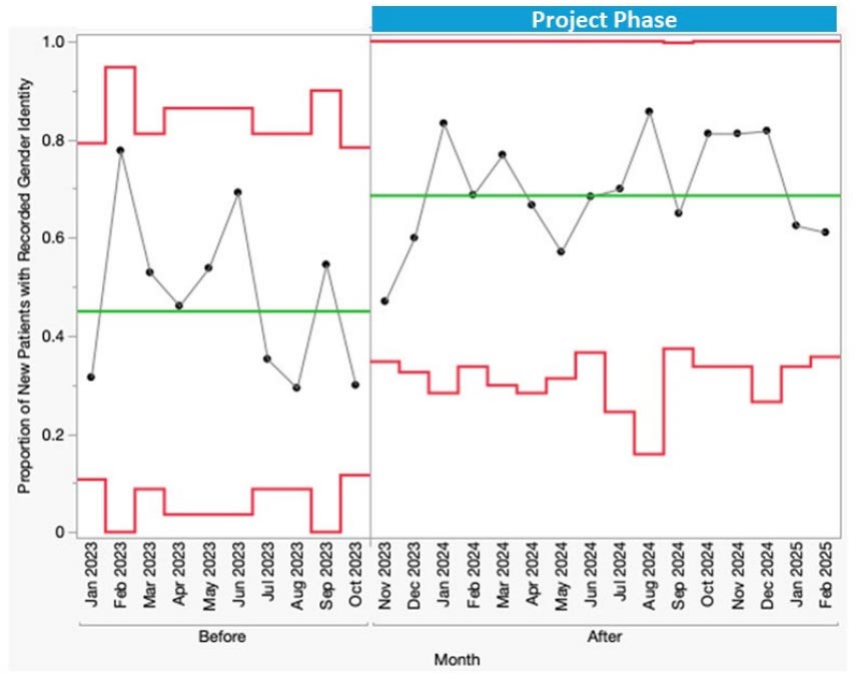
P Chart of Gender Identity Documentation for New Clinic Patients with HIV

**Methods:**

We assessed baseline SOGI documentation rates in our clinic and created a process map to identify SOGI documentation opportunities (Figure 1). In phase one of the project, SOGI data collection was integrated into social work (SW) intake for all new patients. Documentation rates were tracked monthly for 10 months pre-intervention (n=149 patients) and 16 months post-intervention (n=296 patients). Statistical process control charts were used to monitor process improvement. In phase 2 of the project, we met with community partners to assess the acceptability of sending messages via the EHR patient portal to request SOGI information from existing patients.

**Figure 3.**
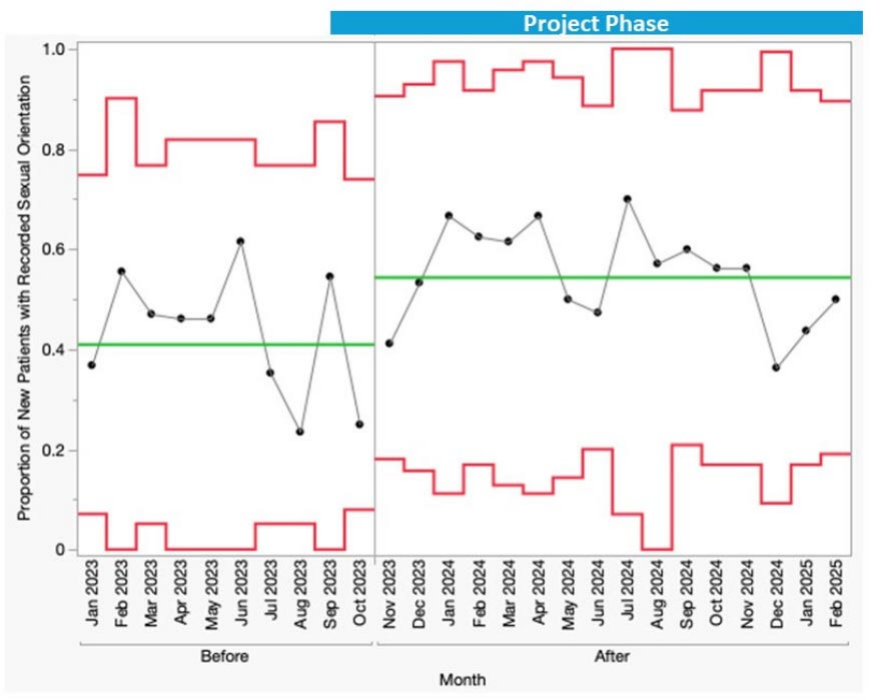
P Chart of Sexual Orientation Documentation for New Clinic Patients with HIV

**Results:**

A total of 316 PWH (avg 14.7 patients/month) were new to clinic during phase one of the study. After implementation of the SW intervention, special cause variation was observed with a sustained shift in the proportion of completed SOGI data fields among new patients. Mean gender identity documentation increased from 45% to 69% (Figure 2). Sexual orientation documentation improved from 41% to 54% (Figure 3). In light of evolving policies impacting privacy and safety for SGM individuals, we elected not to request SOGI information via EHR messages.

**Conclusion:**

Integrating SOGI documentation into the SW workflow for PWH new to clinic led to a sustained improvement in reporting in structured data fields over the first 16 months of our project. To further these efforts, we are creating structured provider note templates to facilitate SOGI documentation for both new and established PWH in the clinic (approximately 2200 patients). For established patients, we are pivoting our approach to ensure this crucial health information is gathered in a trusted and secure manner during provider and SW visits.

**Disclosures:**

All Authors: No reported disclosures

